# Efficiency evaluation of secondary public general hospitals in Shandong Province, China based on three-stage data envelopment analysis

**DOI:** 10.3389/fpubh.2026.1933937

**Published:** 2026-10-08

**Authors:** Shirui Shan, Liang Ma, Tianjiao Zhang, Jingjing Li, Lijun Wang, Wei Xiao, Keke Liu

**Affiliations:** 1 General Office, Affiliated Provincial Hospital of Shandong First Medical University, Jinan, China; 2 Discipline Inspection Commission Office, Affiliated Provincial Hospital of Shandong First Medical University, Jinan, China; 3 Infection Control Office, Affiliated Provincial Hospital of Shandong First Medical University, Jinan, China

**Keywords:** data envelopment analysis, hospitals efficiency, secondary public general hospitals, stochastic frontier analysis, three-stage data envelopment analysis

## Abstract

**Objective:**

This study aimed to evaluate the operational efficiency of secondary-level public general hospitals in Shandong Province, thereby providing scientific evidence for optimising the allocation of hospital healthcare resources.

**Methods:**

A sample of secondary public general hospitals across 16 prefecture-level cities in Shandong Province, China, was selected. The first stage employed the Super-Efficiency SBM model to measure overall technical efficiency (TE), pure technical efficiency (PTE), and scale efficiency (SE). The second stage constructed an SFA regression model to analyse the impact of external environmental factors on input slack variables. The third stage recalculated efficiency using adjusted inputs and original outputs, comparing efficiency differences before and after adjustment alongside regional characteristics.

**Results:**

The TE and PTE of Shandong Province’s secondary public general hospitals topped 1 in the majority of the first stage, while the SE often fell short of 1. The greatest mean values for TE (0.952), PTE (0.983), and SE (0.971) were found in the Lunan Economic Circle. PTE variations were the main factor influencing TE stability, and the provincial capital economic circle (TE = 0.808) and Jiaodong economic circle (TE = 0.851) displayed comparatively lower averages. Second-stage SFA regression results indicate that fiscal allocations positively correlate with hospital practising (assistant) physicians, actual bed capacity, fixed assets, and total cost redundancy (*p* < 0.01), while urbanisation rates negatively correlate with fixed asset redundancy (*p* < 0.01). Following adjustments to input variables in the third stage, TE and PTE for secondary public general hospitals in Shandong Province decreased significantly (TE decreased by 31.93 and 37.16% in provincial capitals and the Jiaodong Economic Zone, respectively), while SE increased slightly.

**Conclusion:**

Secondary-level public general hospitals in Shandong Province demonstrate relatively high overall efficiency, yet remain significantly influenced by external environmental factors, indicating considerable scope for improvement. This study reveals that differentiated improvement strategies should be implemented across distinct regions based on their actual developmental circumstances, thereby propelling hospitals towards high-quality development.

## 1 Introduction

As a developing nation, China continues to increase investment in healthcare resources. Nevertheless, challenges persist regarding the relative scarcity and uneven distribution of healthcare resources nationwide (1). Concurrently, the implementation of new healthcare reform measures, such as reforms to medical insurance payment methods, has compelled Chinese hospitals to continuously strengthen cost control (2). Consequently, the shift in hospital development from ‘scale expansion’ to ‘efficiency-driven growth’ is of paramount importance. China’s public hospitals are categorised into three tiers based on bed capacity: Level I hospitals have fewer than 100 beds, Level II hospitals have between 100 and 500 beds, and Level III hospitals have more than 500 beds (3). As pivotal nodes within the tiered healthcare system, secondary public hospitals are typically situated within China’s county-level administrative regions. They undertake the diagnosis and treatment of common and prevalent illnesses within their catchment areas, alongside the referral of certain complex cases. This effectively safeguards the accessibility, continuity, and equity of primary healthcare services. The Chinese government released the “Notice on Strengthening Performance Assessment of Secondary Public Hospitals” in 2019 to encourage the improvement of operational management in secondary public hospitals. The notice highlighted the necessity of directing secondary public hospitals to consistently improve scientific management and set up a performance assessment system that incorporates operational efficiency. Consequently, assessing the efficiency of secondary public hospitals holds significant importance for optimising healthcare resource utilisation and achieving high-quality development within the public hospital sector.

Shandong Province is a core province within the Bohai Rim Economic Zone, situated along China’s eastern coastline and in the lower reaches of the Yellow River. From 2014 to 2023, Shandong witnessed substantial growth in healthcare resource allocation. The province’s total healthcare expenditure as a percentage of GDP rose from 4.11 to 6.63%. Although the number of public hospitals decreased from 854 to 774, hospital scale continued to expand. The number of hospital beds increased from 297,600 to 400,000, while the number of healthcare professionals increased from 318,000 to 445,800 (4)^,^ representing growth rates of 34.4 and 40.1%, respectively. The emergence of disorderly expansion in some hospitals has not only intensified operational pressures on these institutions but also exacerbated imbalances in resource allocation. Addressing these issues necessitates urgent research into the operational efficiency of public hospitals in Shandong Province, thereby enabling the formulation of differentiated development strategies for hospitals of varying scales.

Currently, a variety of methods exist globally for assessing efficiency within the healthcare sector. Arfa pioneered the combined application of the Free Disposal Hull (FDH) model and non-radial distance functions to evaluate the efficiency of cardiology departments across five Tunisian hospitals (5). However, the FDH model is only suitable for small sample sizes and is sensitive to outliers. Stochastic Frontier Analysis (SFA) can be employed to study hospitals’ technical and cost efficiency (6) offering the advantage of separating random noise and managerial inefficiency components. Nevertheless, its reliability diminishes when data distributions become complex. Additionally, the Translog production function and Stochastic Walk Model may also be utilised for efficiency and marginal benefit assessments (7, 8).

Data Envelopment Analysis (DEA) serves as an effective tool for examining multiple inputs and outputs, and has become the most widely employed method for assessing relative efficiency in recent years. It was first developed jointly by American operations researchers Chames, Cooper, and Rhodes in the early 1980s (9)^.^ The use of a single DEA model to evaluate the efficiency of sample hospitals is relatively common. For instance, Ahmed and Peng employed traditional DEA models to assess the efficiency of public hospitals in Saudi Arabia and China’s H Province, respectively (10, 11), while Afonso utilised a network DEA model to measure the efficiency, quality, and accessibility of nursing services in Portuguese public hospitals (12).

Some scholars have also employed emerging DEA models to investigate efficiency. For instance, Huang combined the DEA-BCC model with the Malmquist index model to analyse the operational performance of 61 tertiary hospitals in Guangxi Province, China (13). Super-efficiency DEA models can facilitate comparative assessments of efficiency scores among hospitals operating within efficient states (14, 15). DEA can also be used in conjunction with Tobit regression to investigate hospital efficiency factors in more detail (16, 17)^.^ However, when determining hospital efficiency, these approaches still fall short of removing the influence of external environmental factors like policy and economic conditions. Furthermore, macro-level regional comparative analysis is lacking in current hospital efficiency research, which primarily uses a micro-level approach to compare efficiency among individual hospitals or hospital categories (18–20). This ignores the effect of regional heterogeneity on efficiency, even though hospital performance is greatly influenced by variables like geographic location, economic development levels, and policy support. Therefore, it is crucial to evaluate hospital efficiency from a regional standpoint in order to suggest tailored efficiency improvement strategies.

Building upon existing research, this study employs a macro-level regional comparative analysis by measuring the efficiency of secondary public general hospitals in Shandong Province’s provincial capital, Jiaodong and Lunan economic zones, and their subordinate prefecture-level cities. It proposes differentiated development strategies based on actual efficiency levels. To lessen the influence of outside variables on hospital inputs, such as policy and economic conditions, the super-efficiency DEA model is paired with SFA regression. By avoiding the impact of random noise and environmental factors in efficiency measurement, this method allows for an accurate assessment of hospital efficiency and offers a quantitative basis for the distinct growth of hospitals across different locations.

## 2 Materials and methods

### 2.1 Data sources

This study examines the efficiency of secondary public general hospitals across different regions, focusing on the provincial capital, Jiaodong and Lunan economic zones, and their respective prefecture-level cities within Shandong Province. The provincial capital economic zone encompasses seven prefecture-level cities: Jinan, Tai’an, Zibo, Dongying, Binzhou, Dezhou, Liaocheng. Before 2019, Laiwu was a city within the provincial capital economic circle. Due to government regional planning, Laiwu was merged into Jinan in 2018. Therefore, we combined the data of Laiwu from 2014 to 2018 with that of Jinan, ensuring that there are 16 geographical units for each year from 2014 to 2023, thus maintaining complete comparability. The Jiaodong Economic Circle comprises five prefecture-level cities: Weifang, Rizhao, Qingdao, Weihai, and Yantai; while the Southern Shandong Economic Circle consists of four prefecture-level cities: Heze, Jining, Zaozhuang, and Linyi (Figure 1).

**Figure 1:**
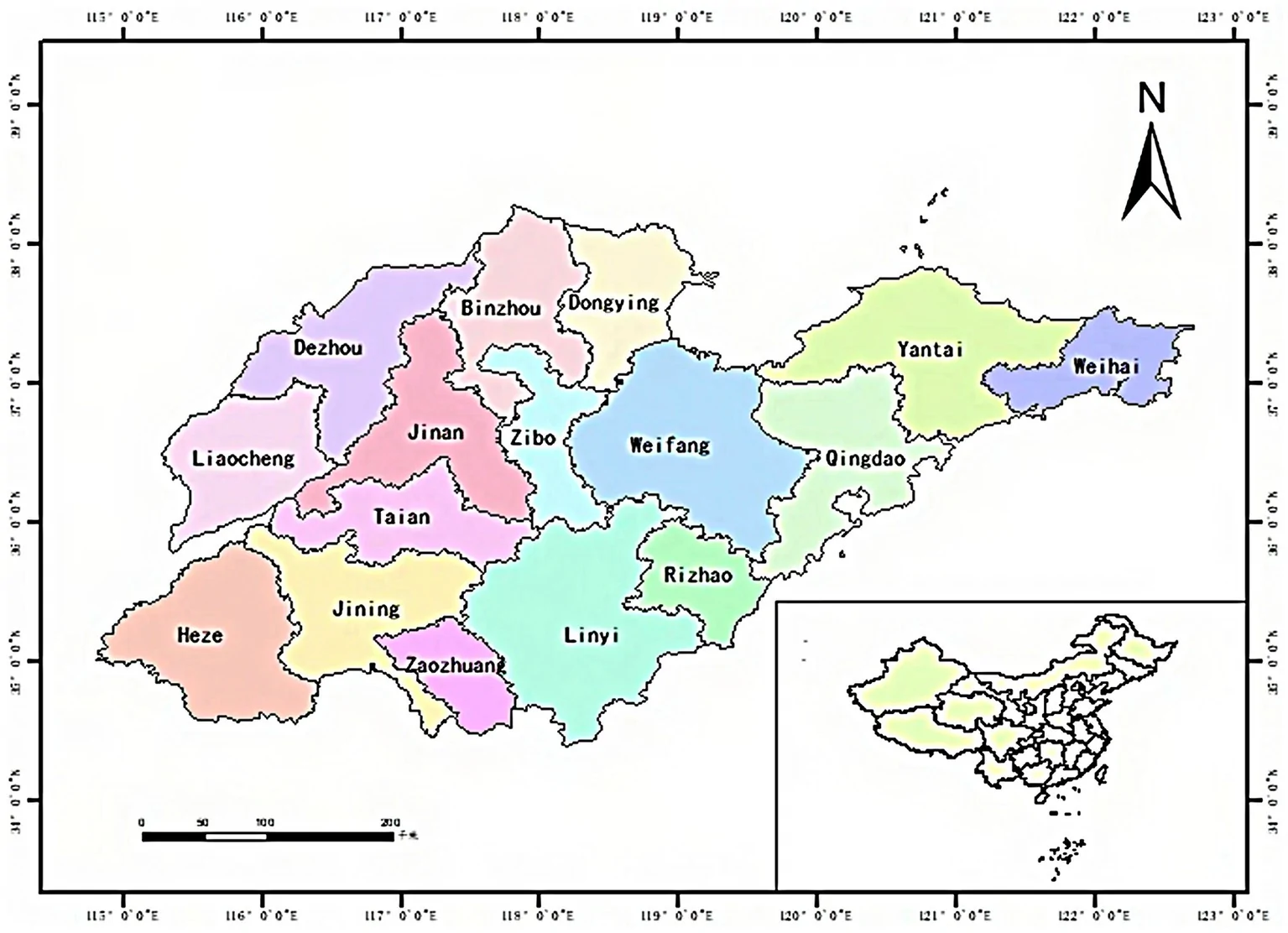
Location distribution map of Shandong Province.

Data sources include relevant operational reports, statistical yearbooks, and health development bulletins from Shandong’s health administrative authorities. This study collected panel data from secondary public general hospitals across Shandong Province and its prefecture-level cities from 2014 to 2023. The three economic zones and respective cities were systematically coded as S1-S3 and H1-H16, respectively.

### 2.2 Input–output indicator screening

First, through a literature review, we systematically organised the input and output indicators employed in recent DEA studies. Input indicators were categorised into three dimensions: human resources, physical resources, and financial resources. Output indicators were classified into two categories: operational and economic. Based on data availability, a database of candidate indicators was established (Table 1).

**Table 1:** Alternative indicator database for hospital efficiency evaluation.

Category of indicator	Evaluation indicators
Input indicators	
Human resources	X1: number of employed staff; X2: number of practising (assistant) physicians
Physical resources	X3: actual number of beds; X4: fixed assets
Financial resources	X5: total expenditure; X6: revenue from government grants
Output indicators	
Operational output	Y1: outpatient and emergency visits; Y2: discharged patients; Y3: total consultations; Y4: bed occupancy rate; Y5: average length of stay
Economic output	Y6: total revenue; Y7: medical revenue

Previous research indicates that the selection of indicator quantity in DEA studies is influenced by the number of decision-making units (DMUs) (21), typically requiring the number of DMUs to exceed twice the sum of input and output indicators. Given the excessive number of indicators in the candidate database, to ensure model validity and stability, this study first conducted a preliminary screening using the coefficient of variation (Table 2), eliminating Y4: bed occupancy rate and Y5: average length of stay. After considering indicator multicollinearity and usage frequency, seven evaluation indicators were ultimately selected (Table 3).

**Table 2:** Coefficient of variation for alternative indicators.

Indicator	Coefficient of variation (CV)	Indicator	Coefficient of variation (CV)
X1	1.93	X2	1.94
X3	1.93	X4	2.01
X5	2.08	X6	1.95
Y1	1.94	Y2	1.94
Y3	1.95	Y4	0.13
Y5	0.14	Y6	1.95
Y7	1.95		

**Table 3:** Final indicators for hospital efficiency evaluation.

Category of indicator	Evaluation indicators
Input indicators	
Human resources	X2: number of practising (assistant) physicians
Physical resources	X3: actual number of beds; X4: fixed assets
Financial resources	X5: total expenditure
Output indicators	
Operational output	Y1: outpatient and emergency visits; Y2: discharged patients;
Economic output	Y6: total revenue;

At the prefecture-level city level, 16 prefecture-level cities are taken as DMUs (2014–2023), with a total of 160 decision-making units; at the level of the three major economic circles, 3 economic circles are taken as DMUs (2014–2023), with a total of 30 decision-making units.

### 2.3 Method

#### 2.3.1 Super-efficient SBM model

Data Envelopment Analysis (DEA) is a technique for comparing the overall technical efficiency (TE) of different decision-making units (DMUs). Compared to traditional DEA models, the super-efficient SBM model can compare DMUs with efficiency values≫1, measuring both excess inputs and shortfalls in outputs, yielding more accurate results (22). This study employs a three-stage DEA approach, utilising Dearun software to construct the Super-Efficiency SBM model. The first stage calculates efficiency using original input and output indicators, while the third stage recalculates efficiency using adjusted input variables and original outputs. The specific evaluation model is as follows:

Assuming n decision-making units (DMUs), each employing m inputs to produce q_₁_ desired outputs while generating q_₂_ undesired outputs, the super-efficiency SBM formula for any DMU is expressed as Equation 1:

$$\min\rho =\frac{1+\frac{1}{m}\sum_{i=1}^{m}\frac{s_{i}^{-}}{x_{\mathrm{ik}}}}{1-\frac{1}{q_{1}+q_{2}}(\sum_{r=1}^{q_{1}}\frac{s_{r}^{+}}{y_{\mathrm{ik}}}+\sum_{t=1}^{q_{2}}\frac{s_{t}^{b-}}{b_{\mathrm{rk}}})}\#$$ (1)

$$s.t.\{\begin{array}{c} \min\theta \\ \sum_{j=1,j\neq k}^{n}\lambda_{j}x_{\mathrm{ij}}-s_{i}^{-}\leq x_{\mathrm{ik}}\;\;\;\;\\ \sum_{j=1,j\neq k}^{n}\lambda_{j}y_{\mathrm{rj}}-s_{r}^{-}\leq y_{\mathrm{rk}}\;\;\;\\ \sum_{j=1,j\neq k}^{n}\lambda_{j}b_{\mathrm{tj}}-s_{t}^{b-}\leq b_{\mathrm{tk}}\;\;\;\\ 1-\frac{1}{q_{1}+q_{2}}(\sum_{r=1}^{q_{1}}\frac{s_{r}^{+}}{y_{\mathrm{ik}}}+\sum_{t=1}^{q_{2}}\frac{s_{t}^{b-}}{b_{\mathrm{rk}}})>0\;\; \end{array}$$

Here, ρ denotes the comprehensive technical efficiency (TE) of the kth DMU, where a higher value indicates greater efficiency. $x_{\mathrm{ik}}$ represents the ith input of the kth DMU, $y_{\mathrm{rk}}$ denotes the rth expected output of the kth DMU, and $b_{\mathrm{tk}}$ signifies the tth non-expected output of the kth DMU; $s_{i}^{-}$ denotes the input slack variable, reflecting the redundancy of input i, i.e., the difference between actual and optimal input; $s_{r}^{+}$ denotes the expected output slack variable, reflecting the deficiency of expected output r, i.e., the difference between optimal and actual output; $s_{t}^{b-}$ denotes the non-expected output slack variable, reflecting the excess value of the tth non-expected output, i.e., the difference between actual output and optimal control quantity; $\lambda_{j}$ represents the weighting coefficient for any DMU other than the kth DMU, used to construct the efficiency frontier surface, embodying the contribution of different DMUs to the efficiency frontier surface.

In addition, TE is calculated based on the SBM model under the CRS assumption. PTE is calculated based on the SBM model under the VRS assumption. SE = TE/PTE. When the super-efficiency is greater than 1, we adopt the Super-SBM model, whose score indicates that the distance between the DMU and the efficient frontier can be greater than 1. At this time, the calculation of SE still maintains the algebraic relationship of SE = TE/PTE, and is no longer limited to the [0,1] interval.

In hospital efficiency measurement, TE can be decomposed into PTE (Pure Technical Efficiency) and SE (Scale Efficiency). PTE measures a hospital’s efficiency in terms of technical proficiency and management capability, i.e., the degree of optimisation in resource allocation and technology application at an ideal scale (disregarding scale effects). When PTE > 1: This indicates that under the current input scale, the hospital’s management level and technology application have reached optimality, with no idle resources; When PTE < 1, it indicates potential deficiencies in pure technical efficiency due to inadequate management capabilities, suboptimal technical proficiency, or imbalanced resource allocation. SE measures the alignment between a hospital’s production scale and its optimal scale, reflecting whether economies of scale are optimally utilised. When SE = 1, it signifies the hospital operates at its optimal scale; reducing or expanding scale would result in efficiency declines. When SE < 1, two scenarios arise: Increasing Returns to Scale (IRS) signifies the hospital is undersized, necessitating expanded inputs such as beds and personnel to boost output; Decreasing Returns to Scale (DRS) indicates the hospital is oversized, with resources overly dispersed leading to underutilisation.

#### 2.3.2 SFA regression model

Slack variables are influenced by managerial inefficiency, environmental factors and random errors, thereby introducing interference into the measurement of hospital input–output and efficiency. This study first employs the input slack variable $s_{\mathrm{ij}}$ as the dependent variable, selecting five environmental variables $Z_{j}\;$ population size, per capita GDP, urbanisation rate, per capita disposable income, and fiscal allocation input—as independent variables. When the regression coefficient for an environmental variable is >0, it indicates that the input slack variable increases with rising environmental variables, leading to input wastage; the opposite holds true. The SFA cost function was constructed using Frontier 4.1 software. The specific model formula is as Equation 2:

$$s_{\mathrm{ij}}=f^{i}(Z_{j};\beta^{i})+\nu_{\mathrm{ij}}+\mu_{\mathrm{ij}},i=1,2,\ldots \ldots ,n\#$$ (2)

Where $Z_{j}=(Z_{1},Z_{2},\ldots \ldots Z_{p})$ , p denotes the number of environmental variables, $f^{i}(Z_{j};\beta^{i})$ represents the influence function of the environmental variable pair on input relaxation, and $\beta^{i}$ is the coefficient of the environmental variable. $\nu_{\mathrm{ij}}+\mu_{\mathrm{ij}}$ denotes the mixed error term, where $\nu_{\mathrm{ij}}$ represents random errors caused by sudden public health events, etc., and $\mu_{\mathrm{ij}}$ denotes managerial inefficiency arising from process redundancy or idle resources, with the two being mutually independent. Parameters $\;\beta^{i}$ , $\alpha^{2}\;$ , and γ can be estimated using the maximum likelihood technique in Frontier4.1, enabling calculation of the proportion of managerial inefficiency. Here, γ denotes the ratio of managerial inefficiency variance to total variance, with the formula $\gamma =\alpha^{2}/(\alpha_{\mu }^{2}+\alpha_{\nu }^{2}).$ A γ value closer to 1 indicates slack variables are primarily driven by managerial inefficiency, while a value closer to 0 suggests random error predominates. Additionally, $\alpha =\sqrt{\alpha_{\mu }^{2}+\alpha_{v}^{2}}$ .

To eliminate interference from environmental factors and random errors on efficiency, the slack variable $s_{\mathrm{ij}}$ is adjusted using the Equation 3:

$$\begin{array}{c} \begin{array}{l} X_{\mathrm{ij}}^{S}=X_{\mathrm{ij}}+[\max\{K_{j}\hat{B_{i}}\}-K_{j}\hat{B_{i}}]+[\max\{\hat{\nu_{\mathrm{ij}}}-\nu_{\mathrm{ij}}\}],\\ i=1,2,\ldots \ldots m;j=1,2,\ldots \ldots ,n\# \end{array} \end{array}$$ (3)

Here, $X_{\mathrm{ij}}^{S}$ denotes the adjusted input variable, ${ℬ}_{i}$ is the estimated value of the environmental variable ${ℬ}_{i},\hat{\nu_{\mathrm{ij}}}$ is the fitted value of the random error *ν*_ij_. $\left[\max\{K_{j}\hat{{ℬ}_{i}}\}-K_{j}\hat{{ℬ}_{i}}\right]$ denotes the elimination of environmental variation effects, i.e., subject all DMUs to a unified external environment and random shocks. $[\max\{\hat{\nu_{\mathrm{ij}}}-\nu_{\mathrm{ij}}\}]\;$ denotes the elimination of random error effects, meaning all DMUs face identical random shocks.

## 3 Results

### 3.1 Analysis of efficiency values in the first-stage super-efficient SBM model

From 2014 to 2023, both TE and PTE scores for secondary public general hospitals in Shandong Province exceeded 1 in eight years, indicating effective performance. SE remained below 1 in all years except 2017 and 2018. Comparing annual data reveals that Shandong’s secondary public general hospitals demonstrated poorer efficiency scores in 2020 and 2022, with both TE and PTE showing significant year-on-year declines. PTE decreased by over 20% in both years, while SE, though remaining below 1, registered slight increases (Table 4).

**Table 4:** Efficiency scores for Stage I of secondary public general hospitals in Shandong Province.

Year	TE	PTE	SE
2014	1.059	1.076	0.984
2015	1.004	1.006	0.999
2016	1.038	1.048	0.990
2017	1.013	1.013	1.000
2018	1.001	1.001	1.000
2019	1.009	1.022	0.987
2020	0.768	0.775	0.991
2021	1.001	1.005	0.996
2022	0.792	0.793	0.999
2023	1.045	1.167	0.896

For Economic Zone S1 from 2014 to 2023, the mean values of TE, PTE, and SE were 0.808, 0.934, and 0.870, respectively. TE exhibited an upward trend, rising from 0.775 to 1.005, PTE showed pronounced fluctuations, while SE generally converged towards 1 but experienced minor fluctuations after 2019. Economic Zone S2 recorded average values of 0.851 for TE, 0.930 for PTE, and 0.921 for SE. TE declined from 0.900 to 0.794,while PTE remained relatively stable except between 2020 and 2022. SE also decreased from 0.899 to 0.793. Economic Zone S3 recorded average values of 0.952 for TE, 0.983 for PTE, and 0.971 for SE. TE primarily exhibited growth, PTE rose rapidly from 0.872 to 1.213, while SE remained relatively stable, approaching optimal scale allocation (Table 5). Comparing the three economic zones reveals that S3 exhibits the highest average efficiency metrics and the fastest growth rate. S1 has the lowest average TE and SE, while S2 has the lowest average PTE.

**Table 5:** First-stage efficiency of secondary public general hospitals in economic circles and prefecture-level cities, Shandong Province.

Area	2014				2015				2016				2017				2018			
	TE	PTE	SE	RTS	TE	PTE	SE	RTS	TE	PTE	SE	RTS	TE	PTE	SE	RTS	TE	PTE	SE	RTS
S1	0.775	1.054	0.735	DRS	0.725	0.877	0.827	DRS	0.793	1.008	0.786	DRS	0.848	1.016	0.835	DRS	0.841	0.957	0.879	DRS
H1	0.631	1.000	0.630	IRS	0.760	1.000	0.760	IRS	0.294	0.420	0.701	IRS	1.007	1.007	1.000	IRS	0.911	1.000	0.911	IRS
H2	0.153	0.469	0.325	IRS	0.139	0.398	0.348	IRS	0.165	0.365	0.451	IRS	0.150	0.299	0.500	IRS	0.147	0.248	0.590	IRS
H3	0.469	1.000	0.469	IRS	0.642	1.000	0.642	IRS	0.794	1.005	0.790	IRS	1.025	1.025	1.000	CRS	0.504	0.620	0.812	IRS
H4	0.197	0.597	0.331	IRS	0.280	0.567	0.494	IRS	0.260	0.496	0.524	IRS	0.310	0.508	0.610	IRS	0.320	0.473	0.676	IRS
H5	0.099	0.283	0.350	IRS	0.129	0.258	0.497	IRS	0.211	0.342	0.617	IRS	0.265	0.375	0.708	IRS	0.212	0.276	0.768	IRS
H6	0.253	0.593	0.426	IRS	0.279	0.509	0.548	IRS	0.356	0.547	0.651	IRS	0.265	0.391	0.678	IRS	0.294	0.386	0.760	IRS
H7	0.195	1.003	0.194	IRS	0.309	0.613	0.504	IRS	0.252	0.439	0.575	IRS	0.279	0.415	0.673	IRS	0.294	0.395	0.744	IRS
S2	0.900	1.001	0.899	DRS	1.003	1.003	1.000	DRS	1.013	1.016	0.997	IRS	1.012	1.014	0.999	DRS	1.005	1.013	0.992	IRS
H8	1.004	1.004	1.000	IRS	1.000	1.000	1.000	IRS	1.005	1.007	0.998	IRS	1.017	1.017	1.000	CRS	0.694	0.804	0.864	IRS
H9	0.109	1.070	0.102	IRS	0.110	0.699	0.157	IRS	0.414	1.006	0.412	IRS	0.498	1.010	0.493	IRS	0.328	0.849	0.387	IRS
H10	0.320	0.830	0.385	IRS	0.325	0.618	0.525	IRS	0.409	0.725	0.564	IRS	0.357	0.561	0.636	IRS	0.356	0.520	0.684	IRS
H11	0.218	1.000	0.218	IRS	0.190	0.327	0.582	IRS	0.196	0.338	0.580	IRS	0.312	0.455	0.686	IRS	0.303	0.410	0.740	IRS
H12	0.070	1.000	0.070	IRS	0.123	0.427	0.289	IRS	0.111	0.282	0.393	IRS	0.349	1.000	0.349	IRS	0.194	1.006	0.193	IRS
S3	0.866	0.872	0.993	IRS	1.037	1.046	0.991	IRS	1.041	1.045	0.996	DRS	1.011	1.013	0.997	IRS	0.910	1.002	0.909	IRS
H13	0.062	0.217	0.287	IRS	0.129	0.279	0.462	IRS	0.350	0.454	0.771	IRS	0.191	0.273	0.700	IRS	0.078	0.120	0.645	IRS
H14	0.164	0.342	0.479	IRS	0.213	0.372	0.572	IRS	0.283	0.413	0.685	IRS	0.167	0.240	0.694	IRS	0.238	0.305	0.782	IRS
H15	0.172	1.056	0.162	IRS	0.168	0.639	0.263	IRS	0.125	0.320	0.390	IRS	0.217	0.412	0.527	IRS	0.157	0.269	0.585	IRS
H16	0.446	1.000	0.446	IRS	0.229	0.427	0.537	IRS	0.407	1.000	0.407	IRS	1.011	1.011	1.000	IRS	0.339	0.443	0.765	IRS
Mean	0.285	0.779	0.367		0.314	0.571	0.511		0.352	0.572	0.594		0.464	0.625	0.703		0.336	0.508	0.682	

Area	2019				2020				2021				2022				2023			
	TE	PTE	SE	RTS	TE	PTE	SE	RTS	TE	PTE	SE	RTS	TE	PTE	SE	RTS	TE	PTE	SE	RTS
S1	0.934	1.030	0.906	DRS	0.735	0.817	0.899	DRS	0.772	0.802	0.964	DRS	0.655	0.695	0.942	DRS	1.005	1.087	0.925	DRS
H1	1.060	1.060	1.000	DRS	1.017	1.017	1.000	CRS	1.146	1.157	0.991	DRS	0.778	0.782	0.995	IRS	1.026	1.027	0.998	IRS
H2	0.207	0.329	0.629	IRS	0.245	0.308	0.795	IRS	0.248	1.001	0.248	IRS	0.313	0.351	0.891	IRS	1.021	1.021	0.999	IRS
H3	0.720	0.764	0.942	IRS	0.564	0.692	0.815	IRS	1.008	1.019	0.989	IRS	0.519	0.555	0.935	IRS	1.241	1.315	0.943	IRS
H4	0.243	0.343	0.709	IRS	0.407	0.506	0.804	IRS	0.315	0.367	0.859	IRS	0.388	0.413	0.939	IRS	0.392	0.401	0.978	IRS
H5	0.382	0.436	0.876	IRS	0.404	0.432	0.936	IRS	0.624	0.625	0.998	IRS	0.574	0.575	0.999	IRS	0.617	0.617	1.000	CRS
H6	0.312	0.369	0.845	IRS	0.357	0.410	0.869	IRS	0.362	0.396	0.914	IRS	0.347	0.353	0.984	IRS	0.315	0.315	1.000	CRS
H7	0.298	0.370	0.806	IRS	0.290	0.334	0.869	IRS	0.312	0.333	0.938	IRS	0.342	0.346	0.990	IRS	0.386	0.386	1.000	CRS
S2	0.839	1.181	0.711	IRS	0.583	0.648	0.900	IRS	0.724	0.744	0.974	IRS	0.641	0.679	0.944	IRS	0.794	1.002	0.793	IRS
H8	0.714	0.933	0.765	IRS	0.576	0.655	0.880	IRS	0.652	0.688	0.948	IRS	0.749	0.790	0.948	IRS	0.698	1.001	0.697	IRS
H9	0.189	0.399	0.474	IRS	0.261	0.449	0.581	IRS	0.338	0.539	0.626	IRS	0.481	0.682	0.705	IRS	0.583	0.948	0.615	IRS
H10	0.323	0.437	0.740	IRS	0.383	0.450	0.851	IRS	0.274	0.328	0.837	IRS	0.281	0.319	0.879	IRS	0.375	0.425	0.880	IRS
H11	0.331	0.403	0.820	IRS	0.539	0.562	0.958	IRS	0.490	0.490	1.000	DRS	0.520	0.521	0.999	DRS	0.479	0.480	0.998	DRS
H12	0.239	1.049	0.228	IRS	0.296	0.438	0.675	IRS	0.410	0.490	0.838	IRS	0.339	0.475	0.713	IRS	0.346	0.532	0.650	IRS
S3	1.011	1.029	0.983	IRS	0.746	0.758	0.985	IRS	1.008	1.010	0.998	DRS	0.840	0.844	0.995	DRS	1.054	1.213	0.868	DRS
H13	0.104	0.144	0.726	IRS	0.166	0.194	0.852	IRS	0.266	0.266	1.000	DRS	0.275	0.275	1.000	CRS	0.363	0.363	0.998	DRS
H14	0.258	0.299	0.862	IRS	0.744	0.745	0.998	IRS	0.605	0.606	0.999	DRS	0.413	0.414	0.999	DRS	0.651	0.653	0.998	DRS
H15	0.153	0.226	0.677	IRS	0.215	0.278	0.774	IRS	0.194	0.226	0.857	IRS	0.257	0.276	0.934	IRS	0.197	0.197	1.000	CRS
H16	0.386	0.405	0.954	IRS	0.536	0.587	0.914	IRS	0.495	0.572	0.865	IRS	1.004	1.004	1.000	CRS	1.065	1.132	0.941	DRS
Mean	0.370	0.498	0.753		0.437	0.504	0.848		0.484	0.569	0.869		0.474	0.508	0.932		0.610	0.676	0.918	

Analysis of the TE values across Shandong Province’s sixteen prefecture-level cities reveals that the mean distribution ranged from 0.186 to 0.863 between 2014 and 2023. with no prefecture-level city exceeding a mean TE value of 1. H1 recorded the highest mean TE value at 0.863, while H15 exhibited the lowest at 0.186. Among these, H1, H8 and H3 recorded relatively high TE averages above 0.7, while H7, H2, H12, H13, and H15 exhibited lower TE averages below 0.3. Regarding PTE, only H1 and H3 among the sixteen prefecture-level cities had a mean PTE exceeding 0.9,while two cities—H13 and H15—had lower mean PTE values below 0.4. Regarding SE, the average values across prefecture-level cities were relatively high,more than half of the cities have SE averages exceeding 0.7,they are H1, H3, H5, H6, H7, H8, H11, H13, H14 and H16 (Table 5). Comparing the efficiency values across prefecture-level cities reveals that the secondary public general hospitals in H1, H8, and H3 demonstrated higher and more balanced efficiency.

Chronologically, TE fluctuations across the three economic zones remained relatively stable between 2014 and 2018, all above 0.8. The fluctuation range of TE in prefecture-level cities is greater than that in the three economic zones. The trend in PTE mirrors that of TE, both achieving high efficiency in 2023. Between 2014 and 2023, the SE mean value was consistently below 1. Analysis of economies of scale revealed that most prefecture-level cities exhibited increasing returns to scale, indicating suboptimal production scale. Notably, SE means were relatively high in 2022 (0.932) and 2023 (0.918) (Table 5).

### 3.2 Analysis of second-stage SFA regression results

The SFA regression results indicate that for the 2014–2023 period, the one-sided likelihood ratio tests for all four input variables exceeded the critical value (*p* < 0.01), confirming the model’s validity. Specifically: Per capita GDP exhibited a negative effect on the number of practising (assistant) physicians (*p* < 0.05) and the number of actual beds (*p* < 0.05),but has a positive impact on total expenditure (*p* < 0.05). Per capita GDP Urbanisation rate showed a negative effect on the number of actual beds (*p* < 0.1) and fixed assets (*p* < 0.01), while its effect on total expenditure was also negative. Per capita disposable income had a significant positive impact on fixed assets (*p* < 0.01). Fiscal allocation maintained a consistently positive and highly significant influence on all four input variables (*p* < 0.01) (Table 6). Overall, fiscal allocation exerted consistently strong influence on the four input-related variables, while the effects of urbanisation rate and per capita GDP gradually shifted over time.

**Table 6:** SFA regression analysis of stage 2 environmental factors on input slack variables.

Environmental factors	Number of practising (assistant) physicians		Actual number of beds		Fixed assets		Total expenditure	
	Coef.	SE	Coef.	SE	Coef.	SE	Coef.	SE
Population	−0.170	0.181	0.400	0.508	−289.108^*^	167.297	−0.131	0.263
Per capita GDP	−0.003^**^	0.002	−0.009^**^	0.004	1.346	1.521	0.003^**^	0.002
Urbanisation rate	−10.479	10.552	−14.066^*^	8.556	−49061.764^***^	2091.757	−23.619^*^	18.931
Per capita disposable income	0.003	0.009	−0.015	0.020	27.273^***^	5.634	−0.011	0.017
Fiscal allocation investment	0.001^***^	0.000	0.002^***^	0.000	0.479^***^	0.032	0.001^***^	0.000
Sigma-squared (σ^2^)	140663.100	5.580	801823.450	1.000	326,263,990,000	1.000	116833.120	14.139
gamma (γ)	0.597	0.049	0.457	0.067	0.624	0.040	0.713	0.050
Log Likelihood	−1121.578		−1282.352		−2307.334		−2095.440	
LR test of the one-sided error	41.713		25.471		38.173		52.354	

*, **, and *** denote *p* < 0.1, *p* < 0.05, and *p* < 0.01, respectively.

### 3.3 Analysis of phase III super-efficiency SBM model results

Phase III super-efficiency SBM model results indicate that TE for Shandong’s secondary public general hospitals exceeded 1 in only 3 years. TE and PTE remained relatively low between 2019 and 2022, ranging from 0.585 to 0.662 and 0.585 to 0.716, respectively. However, SE reached 1 in both 2019 and 2020, signifying that hospitals achieved optimal production scale. Compared with the primary efficiency of Phase I, both TE and PTE declined after input adjustments, with a significant decrease in 2019: TE fell from 1.009 to 0.626 and PTE from 1.022 to 0.626. Nevertheless, SE increased that year from 0.987 to 1 (Table 7).

**Table 7:** Efficiency scores for stage III of secondary public general hospitals in Shandong Province.

Year	TE	PTE	SE
2014	1.038	1.044	0.994
2015	0.981	1.003	0.978
2016	0.969	1.009	0.961
2017	1.018	1.019	0.999
2018	0.905	1.001	0.904
2019	0.626	0.626	1.000
2020	0.585	0.585	1.000
2021	0.662	0.716	0.925
2022	0.650	0.700	0.929
2023	1.036	1.160	0.893

Economic zone S1 recorded mean values of 0.550 for TE, 0.674 for PTE, and 0.847 for SE. TE remained generally low, with PTE showing a declining trend from 2014 to 2022 before rising sharply above 1 in 2023. SE was relatively low in 2019 and 2023, with minimal variation in other years. Economic zone S2 recorded mean values of 0.535 for TE, 0.554 for PTE, and 0.980 for SE. TE and PTE remained low from 2019 to 2023, while SE consistently equalled 1 from 2016 to 2023; Economic zone S3 recorded TE, PTE, and SE values of 0.835, 0.874, and 0.963, respectively. Both TE and PTE began declining from 2019, recovering to higher levels by 2023, whereas SE exhibited a decrease in 2023. Comparing efficiency against original inputs reveals that, after controlling for external environmental impacts, TE declined in Economic Zones S1 and S2 during 2014 and 2023, with average decreases of 32.8 and 36.5%, respectively. TE increased in certain years within Economic Zone S3 (e.g.2014, 2017, and 2023), though the overall trend remained downward (Table 8).

**Table 8:** Second-stage efficiency of secondary public general hospitals in economic circles and prefecture-level cities, Shandong Province.

Area	2014				2015				2016				2017				2018			
	TE	PTE	SE	RTS	TE	PTE	SE	RTS	TE	PTE	SE	RTS	TE	PTE	SE	RTS	TE	PTE	SE	RTS
S1	0.606	0.678	0.895	DRS	0.575	0.622	0.926	DRS	0.622	0.682	0.911	DRS	0.654	0.738	0.887	DRS	0.644	0.753	0.855	DRS
H1	0.718	1.000	0.718	IRS	0.809	1.000	0.809	IRS	0.547	0.663	0.825	IRS	0.952	1.000	0.952	IRS	0.946	1.000	0.946	IRS
H2	0.365	0.626	0.583	IRS	0.284	0.418	0.681	IRS	0.288	0.355	0.811	IRS	0.289	0.317	0.910	IRS	0.300	0.308	0.974	IRS
H3	0.258	0.454	0.568	IRS	0.254	0.338	0.752	IRS	0.255	0.309	0.825	IRS	0.284	0.314	0.903	IRS	0.262	0.284	0.923	IRS
H4	0.292	0.584	0.500	IRS	0.292	0.410	0.712	IRS	0.291	0.386	0.756	IRS	0.314	0.380	0.826	IRS	0.324	0.352	0.922	IRS
H5	0.381	0.582	0.654	IRS	0.381	0.485	0.787	IRS	0.432	0.514	0.841	IRS	0.468	0.520	0.899	IRS	0.459	0.503	0.913	IRS
H6	0.388	1.000	0.388	IRS	0.404	0.526	0.769	IRS	0.451	0.545	0.827	IRS	0.415	0.448	0.927	IRS	0.427	0.448	0.954	IRS
H7	0.279	1.000	0.279	IRS	0.317	0.429	0.738	IRS	0.338	0.395	0.857	IRS	0.372	0.394	0.945	IRS	0.391	0.402	0.972	IRS
S2	0.845	1.012	0.835	DRS	0.802	0.829	0.968	DRS	0.644	0.644	1.000	CRS	0.629	0.629	1.000	CRS	0.570	0.570	1.000	CRS
H8	0.630	1.000	0.630	IRS	0.704	1.000	0.704	IRS	0.672	1.000	0.672	IRS	0.714	0.853	0.837	IRS	0.728	0.772	0.943	IRS
H9	0.205	0.760	0.270	IRS	0.180	0.353	0.511	IRS	0.207	0.305	0.680	IRS	0.217	0.293	0.741	IRS	0.149	0.184	0.813	IRS
H10	0.501	1.000	0.501	IRS	0.473	0.695	0.680	IRS	0.374	0.492	0.761	IRS	0.400	0.474	0.845	IRS	0.366	0.397	0.920	IRS
H11	0.515	1.000	0.515	IRS	0.475	0.619	0.767	IRS	0.403	0.495	0.814	IRS	0.424	0.463	0.914	IRS	0.421	0.439	0.958	IRS
H12	0.208	1.000	0.208	IRS	0.218	0.444	0.491	IRS	0.263	0.377	0.699	IRS	0.197	1.000	0.197	IRS	0.131	0.186	0.706	IRS
S3	0.881	0.881	1.000	CRS	0.932	0.946	0.985	IRS	1.009	1.014	0.995	IRS	1.033	1.037	0.996	DRS	0.856	1.007	0.849	IRS
H13	0.358	1.000	0.358	IRS	0.391	1.000	0.391	IRS	0.447	1.000	0.447	IRS	0.460	0.532	0.865	IRS	0.328	0.347	0.947	IRS
H14	0.457	0.674	0.677	IRS	0.474	0.615	0.771	IRS	0.466	0.605	0.771	IRS	0.473	0.553	0.855	IRS	0.514	0.572	0.898	IRS
H15	0.195	1.000	0.195	IRS	0.206	0.459	0.449	IRS	0.230	0.299	0.767	IRS	0.233	0.273	0.852	IRS	0.224	0.238	0.942	IRS
H16	0.611	1.000	0.611	IRS	0.534	0.759	0.704	IRS	0.560	1.000	0.560	IRS	0.620	1.000	0.620	IRS	0.559	0.638	0.877	IRS
Mean	0.398	0.855	0.478		0.400	0.597	0.670		0.389	0.546	0.745		0.427	0.551	0.818		0.408	0.442	0.913	

Area	2019				2020				2021				2022				2023			
	TE	PTE	SE	RTS	TE	PTE	SE	RTS	TE	PTE	SE	RTS	TE	PTE	SE	RTS	TE	PTE	SE	RTS
S1	0.514	0.779	0.660	DRS	0.443	0.474	0.936	DRS	0.442	0.476	0.929	DRS	0.425	0.452	0.939	DRS	0.573	1.087	0.528	DRS
H1	1.040	1.052	0.989	IRS	1.016	1.017	0.999	IRS	1.075	1.076	0.999	IRS	0.770	0.770	1.000	DRS	0.810	1.002	0.808	IRS
H2	0.326	0.326	1.000	CRS	0.315	0.315	1.000	CRS	0.342	0.342	1.000	IRS	0.360	0.360	1.000	DRS	0.489	0.489	1.000	IRS
H3	0.300	0.314	0.955	IRS	0.281	0.291	0.963	IRS	0.305	0.317	0.963	CRS	0.274	0.285	0.960	CRS	0.363	0.375	0.969	CRS
H4	0.250	0.268	0.932	IRS	0.239	0.262	0.911	IRS	0.267	0.279	0.957	IRS	0.275	0.289	0.951	IRS	0.429	0.430	0.998	IRS
H5	0.549	0.573	0.957	IRS	0.553	0.561	0.985	IRS	0.686	0.688	0.998	IRS	0.666	0.666	1.000	IRS	0.729	0.729	1.000	IRS
H6	0.457	0.468	0.976	IRS	0.377	0.377	1.000	CRS	0.353	0.357	0.991	CRS	0.420	0.420	1.000	CRS	0.445	0.445	1.000	CRS
H7	0.407	0.409	0.995	IRS	0.401	0.401	0.999	IRS	0.433	0.433	1.000	IRS	0.430	0.430	1.000	CRS	0.476	0.476	1.000	DRS
S2	0.315	0.315	1.000	CRS	0.327	0.327	1.000	CRS	0.424	0.424	1.000	CRS	0.370	0.370	1.000	CRS	0.424	0.424	1.000	CRS
H8	0.457	0.459	0.996	IRS	0.383	0.388	0.987	IRS	0.463	0.467	0.992	DRS	0.392	0.392	1.000	DRS	0.320	0.328	0.976	IRS
H9	0.153	0.174	0.879	IRS	0.153	0.165	0.923	IRS	0.250	0.257	0.973	CRS	0.249	0.265	0.941	CRS	0.253	0.268	0.946	CRS
H10	0.375	0.382	0.981	IRS	0.391	0.391	1.000	CRS	0.376	0.376	1.000	IRS	0.373	0.373	1.000	IRS	0.453	0.453	1.000	IRS
H11	0.434	0.440	0.986	IRS	0.635	0.635	1.000	CRS	0.665	0.667	0.998	IRS	0.681	0.681	1.000	IRS	0.646	0.646	1.000	IRS
H12	0.138	0.187	0.738	IRS	0.193	0.219	0.881	IRS	0.341	0.348	0.979	CRS	0.223	0.240	0.927	IRS	0.230	0.252	0.911	DRS
S3	0.567	0.567	1.000	CRS	0.562	0.562	1.000	CRS	0.670	0.691	0.970	DRS	0.739	0.770	0.959	DRS	1.103	1.260	0.875	DRS
H13	0.342	0.353	0.970	IRS	0.405	0.416	0.975	IRS	0.556	0.557	0.999	IRS	0.525	0.525	1.000	CRS	0.608	0.608	1.000	CRS
H14	0.546	0.589	0.927	IRS	0.872	0.875	0.996	IRS	0.778	0.778	1.000	IRS	0.648	0.648	1.000	CRS	0.796	0.799	0.997	CRS
H15	0.239	0.245	0.978	IRS	0.231	0.237	0.972	IRS	0.277	0.279	0.996	IRS	0.258	0.263	0.982	IRS	0.280	0.282	0.993	IRS
H16	0.522	0.568	0.918	IRS	0.544	0.551	0.988	IRS	0.496	0.496	1.000	IRS	1.006	1.012	0.993	CRS	1.127	1.132	0.995	DRS
Mean	0.408	0.426	0.949		0.437	0.444	0.974		0.479	0.482	0.990		0.472	0.476	0.985		0.528	0.545	0.975	

Comparing prefecture-level cities, H1 exhibited the highest average TE from 2014 to 2023 at 0.868. TE remained above 0.6 in H1, H14, and H16, while 5 cities—H3, H4, H9, H12 and H15—registered lower TE below 0.3. The three prefecture-level cities with the highest PTE averages were H16, H1, and H14, with H1 recording the highest PTE at 0.958. The PTE values for H2, H3, H4, H9, and H15 were lower, below 0.4. Tow prefecture-level cities recorded SE averages exceeding 0.9: H1and H5,while H12 had lower SE averages below 0.7. Compared with the original input, the average technical efficiency (TE) values of all prefecture-level cities have slightly decreased overall. Among them, The TE rankings of H13, H14, H5, and H11 have significantly improved, while H3, H8, and H9 have significantly lagged behind (Table 8).

From a yearly perspective, the average TE values of the three economic circles and prefecture-level cities were at a relatively low level from 2018 to 2019, and began to slowly recover from 2020 onwards. The overall trend of PTE was similar to that of TE. The mean value of PTE was significantly lower from 2018 to 2022 compared to other years, reaching its lowest point of 0.426 in 2019. While the temporal variation of SE was relatively stable, fluctuating between 0.478 and 0.990, with the highest value of 0.990 occurring in 2021 (Table 8).

## 4 Discussion

This study finds that, after controlling for external environmental factors such as population, policy, and economic conditions, the overall TE and PTE of secondary public general hospitals in Shandong Province are relatively high, indicating sound hospital operations. However, the SE of most prefecture-level cities remained below 1 between 2014 and 2023, with economies of scale predominantly exhibiting increasing returns. This suggests that secondary public general hospitals in Shandong have yet to reach the efficiency frontier, and that expanding inputs appropriately based on current scale could significantly increase outputs.

SFA regression results indicate that fiscal allocation, representing policy factors, significantly influences resource allocation across secondary public general hospitals. Economic factors such as urbanisation rate, per capita disposable income, and per capita GDP exert greater influence on the allocation of physical resources like hospital beds and fixed assets. After controlling for external environmental factors affecting input slack variables, TE and PTE values for provincial capitals, the Jiaodong and Lunan economic zones, and prefecture-level cities all decreased substantially, while SE showed a slight increase. This indicates that external environmental factors such as population, policy, and economic conditions significantly influence hospital operations and resource allocation, consistent with George’s findings (23), suggesting considerable room for improvement.

The study found that between 2014 and 2023, the distribution ranges for TE, PTE, and SE in Shandong’s secondary public general hospitals were concentrated between 0.60–1.10, 0.60–1.20, and 0.85–1.00, respectively. After controlling for environmental factors affecting inputs, the concentrated ranges for TE, PTE, and SE were 0.18–0.85, 0.25–0.90, and 0.75–1.00, respectively. In most years, PTE and TE exhibited positive correlations, while SE and TE showed negative correlations. Therefore, considering both trend patterns and distribution ranges, PTE exerts a more pronounced influence on TE. This indicates that internal operational management factors—such as technical proficiency, managerial capability, and resource allocation—are pivotal to enhancing efficiency in Shandong’s secondary public general hospitals. This aligns with Pan’s (24) findings, as secondary public hospitals primarily provide basic diagnostic and therapeutic services to local residents. With relatively stable staffing levels and equipment configurations, hospital scale is also strictly regulated by health authorities. Consequently, operational management factors like improving technical standards and optimising resource allocation exert a more pronounced driving force on efficiency.

Secondly, PTE and TE were significantly lower during the period from 2019 to 2022 compared to other years, while SE saw a slight increase. This trend temporally coincides with the main impact period of the COVID-19 pandemic. During this period, hospitals may have disrupted their regular operational management due to their primary responsibility for epidemic prevention and control tasks. Lagging information technology applications and limited medical service capabilities may also be potential factors contributing to the significant decline in PTE (25). Furthermore, by comparing efficiency across different economic zones and prefecture-level cities, this study reveals pronounced regional disparities among Shandong’s secondary public general hospitals. The provincial capital economic zone exhibits an overall increase in TE, with SE stabilising but neither reaching efficient status. The persistent diminishing returns to scale indicate that secondary public general hospitals in this zone have yet to achieve optimal production scale, reflecting inadequate levels of refined management. Within the Jiaodong Economic Circle, PTE and SE remained relatively stable. However, after controlling for environmental factors affecting input slack variables, overall efficiency declined. This indicates that secondary public hospitals in the Jiaodong Economic Circle exhibit high dependence on external factors such as policy subsidies and regional economic advantages, coupled with poor operational stability and risk resilience, reflecting inadequate self-development capabilities. The Lunan Economic Circle exhibits rapid increases in TCE and PTE alongside a significant decline in SE, with diminishing returns to scale observed throughout 2021–2023. This indicates excessive scale expansion among secondary public general hospitals in the Lunan Economic Circle, exceeding the support capacity of current technological and managerial capabilities.

Our study has certain limitations: as it exclusively examines secondary public general hospitals, we cannot formulate subsequent reform recommendations for other tiers or types of hospitals. Nevertheless, our research remains valuable for enhancing operational management within secondary public hospitals. Future studies could evaluate the efficiency of additional hospital tiers and types. Secondly, while we collected panel data from 2014 to 2023 to evaluate hospital efficiency at different points in time, we did not conduct a dynamic analysis of the causes behind efficiency changes across periods. Future research could integrate other evaluation models for a more comprehensive study. Thirdly, our focus solely on hospitals within Shandong Province limits the generalizability of our research findings. This limitation is common in efficiency studies, and it remains uncertain whether the results can be extended to other provinces or the entire country. Therefore, the conclusions are more suitable as references in specific contexts rather than generalizations.

## 5 Conclusion

This study reveals substantial scope for efficiency improvements within secondary public general hospitals in Shandong Province, with significant regional disparities in performance. Public health emergencies also impact routine hospital operations and management. Addressing current challenges, the research proposes several strategies for relevant government bodies and hospital administrators.

Improvements in internal management factors demonstrably enhance efficiency at primary-level hospitals such as those at county and district levels. Hospitals may therefore advance management upgrades and technological innovation, implement one-stop services to promote digitalisation and standardisation of management processes, and leverage AI tools to optimise the utilisation of technical resources, thereby elevating hospital efficiency and service capacity.

Primary-level hospitals in different development regions may adopt differentiated strategies: Hospitals in economically developed areas should leverage regional resource advantages, prioritise technological innovation, and continuously enhance competitiveness through smart diagnostics and refined management. This reduces reliance on external factors, facilitating a shift from passive adaptation to proactive development. Hospitals in economically underdeveloped regions should prioritise improving resource utilisation efficiency. They should strengthen cooperation and exchange with larger hospitals, leveraging medical consortiums and healthcare community platforms to address talent and technological shortcomings, thereby achieving simultaneous enhancement of hospital capabilities and efficiency.

Administrative bodies should also provide multi-dimensional policy support, particularly for hospitals in areas lacking medical resources. This includes establishing specialised financial subsidies, implementing targeted talent cultivation programmes, and building resource-sharing platforms to help primary-level hospitals reduce operational costs and enhance efficiency.

## Data Availability

The raw data supporting the conclusions of this article will be made available by the authors, without undue reservation.
